# Individual‐Level Interventions for Alcohol‐Related Harm in Night‐Time Entertainment Precincts: A Scoping Review

**DOI:** 10.1111/dar.70244

**Published:** 2026-08-25

**Authors:** Joanne Papageorgiou, Carleen Thompson, Molly McCarthy, Dominique de Andrade

**Affiliations:** ^1^ School of Criminology and Criminal Justice, Griffith University Brisbane Australia; ^2^ Griffith Criminology Institute, Griffith University Brisbane Australia; ^3^ Alfred Deakin Institute for Citizenship and Globalisation Melbourne Australia; ^4^ School of Psychology, Deakin University Geelong Australia; ^5^ School of Psychology, University of Queensland Brisbane Australia

**Keywords:** alcohol, harm, individual‐level intervention, nightlife, scoping review

## Abstract

**Issues:**

Alcohol‐related harm in Night‐Time Entertainment Precincts (NEP) presents significant challenges for public health, law enforcement and policymakers. While population‐wide measures are well established, less attention has been given to individual‐level interventions targeting harmful alcohol consumption and violence. This review evaluates the current evidence base on individual‐level interventions designed to reduce alcohol‐related harm in NEPs.

**Approach:**

A systematic search was conducted in March 2024 (updated search in January 2025) across ProQuest, CINAHL, PsycINFO, Medline, Scopus and grey literature sources (Google Web and Google Scholar). Studies were included if they were peer‐reviewed, published in English and examined the effectiveness/perceived effectiveness of individual‐level interventions for alcohol‐related harm in NEPs.

**Key Findings:**

The search identified 3127 articles, with 15 meeting inclusion criteria. Studies were categorised into exclusion strategies (patron bans; *n* = 10) and behavioural strategies (personalised screening, brief interventions, breathalysers in licensed premises and a bystander intervention; *n* = 5). Outcomes included assault rates, desistance, blood alcohol levels, self‐reported number of drinks consumed and frequency of staff intervention in a bar setting. Evidence on the overall effectiveness of exclusion and behavioural interventions was difficult to assess due to different outcome measures, variability in methodologies and intervention implementation.

**Implications:**

Rigorously designed studies with consistent outcome measures are needed to strengthen the evidence base and increase confidence in the effectiveness of individual‐level interventions that target alcohol‐related harm in NEPs.

**Conclusion:**

Exclusion strategies demonstrated limited evidence supporting their long‐term effectiveness in reducing alcohol‐related harm, whereas behavioural approaches show greater promise for reducing alcohol‐related harm among high‐risk drinkers.

## Key Point Summary

- This review is the first systematic scoping review to assess the effectiveness of individual‐level interventions designed to address alcohol‐related harm in Night‐Time Entertainment Precincts.
- The review highlights the lack of evidence on individual‐level interventions, despite their potential to address the specific risk factors associated with high‐risk individuals who are responsible for a disproportionate level of violence.
- Findings suggest that while some behavioural strategies show promise, the overall evidence base is limited by inconsistent measurement of outcomes and methodological weaknesses, particularly in exclusion‐focused interventions.

## 1 Introduction

Excessive alcohol consumption is a significant public health concern, contributing to a range of physiological and social harms [1]. Night‐Time Entertainment Precincts (NEP), areas with a high concentration of late‐night venues such as bars and clubs, are hotspots for violence and excessive alcohol consumption, exposing individuals to heightened risk of alcohol‐related assault and injury [2]. The risk of experiencing a violent incident increases when a greater number of locations are visited within a NEP [3]. In New South Wales, Australia, licensed premises accounted for the highest number of non‐domestic alcohol‐related assaults in 2025 compared to residential, outdoor, business and public transport locations [4]. Beyond violence, excessive alcohol consumption is associated with a broad range of acute harms, including injury, alcohol poisoning, risky sexual behaviour as well as chronic issues such as long‐term disease and premature death [5]. Excess harms generated within NEPs have implications for health and justice systems, contributing to increased ambulance attendances [6], emergency department presentations and hospitalisations [7, 8].

Efforts to reduce alcohol‐related harm in NEPs have largely focused on population‐level interventions—regulatory strategies, implemented by the government or public health agencies, to improve health outcomes and reduce harm across a whole population. This can include measures such as licensing restrictions, lockout laws, responsible service of alcohol policies, increased police presence and safe‐night spaces or services [9, 10]. Over the past two decades in Australia, population‐level interventions have been implemented to address alcohol‐related harm in entertainment precincts. An early, influential intervention in Newcastle, New South Wales, introduced earlier closing hours among other measures in licensed premises, leading to a 37% reduction in police‐recorded assaults [11], which was sustained over 7 years [12, 13]. Two of Sydney's central entertainment precincts adopted 1:30 am lockout laws (venue entry cut‐offs) and 3:00 am last drinks in 2014, resulting in a 13.3% reduction in non‐domestic assaults [14, 15] and reduced assault‐related hospital presentations, sustained over 5 years [16].

In Queensland, a 3 am lockout was introduced in Surfers Paradise in 2004. An evaluation using police and ambulance records showed there were no significant reductions in crime, violence, head and neck injuries or overdose/intoxication in the 2 years that followed [17]. In 2016, the ‘Tackling Alcohol‐Fuelled Violence’ policy was introduced across 15 designated late‐night entertainment precincts (Safe Night Precincts), incorporating reduced trading hours, bans on high‐alcohol rapid intoxication drinks after midnight, and the implementation of mandatory network identification (ID) scanners within late‐night licensed venues [18]. The Queensland Alcohol‐related Violence and Night‐Time Economy Monitoring evaluation noted promising reductions in some key measures, including ambulance call outs statewide (11% per month on average during 3–6 AM); however, levels of alcohol consumption and harm remained relatively high [2]. A number of studies followed Queensland Alcohol‐related Violence and Night‐Time Economy Monitoring, extending the follow‐up periods to report meaningful reductions in alcohol‐related assaults [19], small impacts in injury presentations to emergency departments [20] and ambulance call‐outs in target precincts [6].

International approaches to addressing alcohol‐related harm in nightlife settings are also dominated by population‐level strategies. For example, Sweden's Stockholm Prevents Alcohol and Drug Problems program combined community mobilisation, responsible beverage service training for bar staff and stricter enforcement of alcohol laws, which resulted in a 29% reduction of violent crime over a 10‐year period in Stockholm's nightlife districts [21]. Alternatively, Cardiff's Violence Prevention Model, developed in the United Kingdom (UK), involves inter‐agency data sharing through the systematic sharing of emergency department injury data with public agencies to inform and guide strategies for reducing alcohol‐related harm [22]. The model has been adopted in several countries, including the United States and Australia [23, 24].

Multi‐pronged interventions in night‐life settings have demonstrated harm reduction effects at a population level [2, 11]; however, public support for these types of interventions has diminished in recent years [25]. Several factors may explain this shift. The significant social and economic impacts of COVID‐19 have led to the relaxation of liquor licensing regulations in many major cosmopolitan cities internationally (e.g., Sydney, Amsterdam) [26, 27], the development of night‐time strategies or implementation plans [28, 29], and the appointment of Nightlife Commissioners and Majors [30] to revitalise nightlife precincts. In addition, multi‐component interventions do not often directly target the small sub‐groups of individuals more likely to contribute to a disproportionate level of violent offending [31, 32]. This highlights the potential for population‐level approaches in NEPs to overlook patrons at greater risk of harm or offending.

Although population‐level interventions appear to reduce overall rates of alcohol‐related harm in NEPs, individuals at higher risky drinking levels, including those whose drinking patterns are more entrenched, may be less responsive to these approaches. A smaller proportion of persistent, high‐risk individuals may require more intensive or specialised interventions [33, 34]. This limitation highlights the importance of developing targeted, individual‐level approaches for addressing high‐risk groups.

Individual‐level interventions are strategies designed to directly influence the attitudes, motivations or behaviours of high‐risk individuals [35]. In the context of violence and heavy alcohol use in NEPs, these interventions typically focus on those most at risk (high‐risk) of engaging in or being affected by such behaviours. These strategies aim to encourage safer choices and reduce harmful outcomes by working with individuals, rather than changing the broader environment or population associated with violence and heavy alcohol use. Examples of individual‐level interventions in these settings include patron banning, which primarily aims to modify an individual's behaviour by restricting entry to NEPs for specified periods, or personalised, brief interventions (BI) which focus on changing the underlying attitudes, beliefs or motivations that drive risky alcohol‐related behaviour.

Exclusion‐based strategies, such as patron banning, are a deterrence‐based strategy for sanctioning and deterring violent or disorderly behaviour [36]. They comprise notices issued by police, courts or venues to temporarily or permanently exclude individuals from licensed premises or NEPs, due to violent or disruptive behaviour. Their purpose is to prevent a banned individual from repeating their disruptive behaviour (specific deterrence) and signal to others the consequences of such behaviour (general deterrence) [37]. Patron banning has become increasingly popular in countries such as Australia and the UK. In the Australian context, this can be attributed to investment in technology‐based surveillance systems, including mandatory ID scanners in licensed venues, which have facilitated the expanding use of banning mechanisms [38]. Although there is recent research examining the type of offences associated with patron banning recipients [39], few studies evaluate the effectiveness of patron banning in reducing harm [40]. There is also marked variation in their duration across jurisdictions [33].

Behavioural strategies, in comparison, aim to facilitate individual‐level change through approaches such as personalised feedback, motivational interviewing (MI) and BI. Often delivered in healthcare settings, BI have been shown to reduce harmful alcohol use and related harms by helping individuals recognise their risky drinking behaviours and encourage safer consumption patterns [41]. Personalised screening and feedback provide tailored insights based on drinking habits [42], and MI fosters intrinsic motivation for change [43]. Screening tools like the Alcohol Use Disorders Identification Test (AUDIT) are commonly used in conjunction with BI to provide personalised feedback and aim to reduce hazardous drinking and its associated risks [44]. In some locations, venue staff have also been trained in bystander awareness training to recognise and intervene when witnessing early signs of alcohol‐related harm, including sexual assault [45]. Bystander awareness training has been shown to change people's attitudes and increase willingness to intervene in situations involving violence, intoxication or unsafe drinking practices in alternative settings such as college campuses [46].

Although population‐level responses to alcohol‐related harm in NEPs have established a reasonable evidence base [10], the impact of individual‐level interventions in this space remains relatively underexplored. Research investigating the effectiveness of individual‐level strategies within NEPs is fragmented, making it difficult to assess whether these strategies are effective. This scoping review synthesises the current evidence on individual‐level interventions that address alcohol‐related harm in NEPs, summarising their effectiveness, implementation challenges, and gaps in the literature. By mapping available research, this review provides a foundation to support the development of further refined and targeted interventions to minimise alcohol‐related harm in nightlife settings.

## 2 Methods

### 2.1 Search Strategy

A comprehensive search strategy was developed to identify interventions and strategies in the literature aimed at reducing alcohol‐related harm in NEPs, with a particular focus on interventions that target high‐risk individuals. High‐risk individuals were defined as those most likely to engage in alcohol‐related harm, including people who exhibit violent behaviour or engage in problematic or risky drinking behaviour. This includes strategies aimed at directly altering the behaviour and/or outcomes of high‐risk individuals, rather than approaches designed to target the broader population.

Scoping reviews are well‐suited for exploring areas with limited research, allowing for a broad mapping of existing evidence and identifying gaps in knowledge about the effectiveness of interventions [47]. This methodology was chosen due to the relatively narrow topic, alcohol‐related harm in NEPs, and to highlight gaps in our understanding of the effectiveness and perceived effectiveness of individual‐level interventions. No existing review to date has directly addressed this study's focus, though several have examined related or adjacent issues within the night‐time economy [9, 10]. The scoping review was developed in accordance with the Joanna Briggs Institute methodological guidelines [48], and a protocol was developed and uploaded to the Open Science Framework (https://osf.io/6fsrm).

A keyword search strategy was developed using terms related to alcohol‐related offending and harm in NEPs. Four main categories of search terms were incorporated using a Boolean search strategy to ensure comprehensive retrieval of relevant literature:

1. (Alcohol OR liquor OR intoxic* OR drink* OR drunk OR beverage OR binge) AND
2. (Violen* OR assault* OR aggress* OR arrest* OR harm*) AND
3. (Polic* OR intervention OR measure OR strateg* OR approach OR “patron ban*” OR law enforcement OR treatment OR program OR ban* OR order OR medical OR “evidence‐based” OR evaluat*) AND
4. (Night* OR precinct* OR premise* OR licence* OR bar OR entertainment OR venue OR district OR nightlife OR night‐life OR nighttime OR night‐time OR nightclub OR night‐club OR late‐night OR pub OR club).

Five major databases relevant to the field were searched using this search strategy: Proquest, CINHAL, PsychINFO, Medline and Scopus. An initial search was conducted on 25 March 2024 for all records published until that date. An updated search was conducted on 16 January 2025 to incorporate any new articles that may be relevant. To ensure comprehensive coverage, a grey literature search was conducted, which involved screening the first 10 pages of results from both Google and Google Scholar. Additionally, citation tracking and forward and backward snowballing techniques were employed to identify further relevant studies.

### 2.2 Eligibility Criteria

Studies were eligible for inclusion if they were peer‐reviewed and published in English. There was no restriction on publication date. Exclusion criteria were established to ensure articles were appropriate for the aims of the review. We applied a modified PICO framework (Population, Intervention, Comparison, Outcome) excluding the comparison element as this review was exploratory in nature, examining the effectiveness of individual interventions rather than comparing them against one another (see Table 1). This is consistent with the original definition of the framework in which the comparison element was described as conditional and only included where relevant [49]. Peer‐reviewed studies published in English and focused on human populations were included, whereas protocols, discussion papers and reviews were excluded. The review targeted interventions in or around licensed premises within NEPs, excluding studies from other alcohol‐present environments such as festivals or private parties.

**TABLE 1:** PIO model.

Category	Include	Exclude
*Population*	Individual interventions in and/or around licensed premises in NEPs.	Other social environments where alcohol is present (e.g., festivals, parties).
*Intervention*	Interventions delivered at the individual level, targeting at‐risk individuals who engage in problematic drinking behaviours in drinking environments. Interventions that aim to reduce alcohol‐related harm (violence, injury, risky drinking or offending behaviour).	Multi‐component, hybrid policies or interventions aimed at targeting whole populations (e.g., alcohol tax/sales, lockout laws, responsible service of alcohol). Interventions addressing problematic alcohol use in settings external to NEPs or other drug use
*Outcome*	Evaluating the effectiveness (or perceived effectiveness) of an individual‐level intervention addressing alcohol‐related harms (including violence, injury, risky drinking or offending behaviour). Perceived effectiveness includes perceptions of an intervention's impact on harm, deterrence, displacement and enforcement/compliance.	Studies discussing the opinions of stakeholders on an intervention (without evaluating its effectiveness or discussing alcohol‐related harm)

Abbreviation: NEP, Night‐Time Entertainment Precinct.

For the purpose of this review, individual‐level interventions are defined through a public health lens, as targeted strategies that aim to directly influence the behaviour, decisions, or treatment outcomes of specific at‐risk individuals [50], specifically those who engage in risky alcohol behaviour and/or consumption in NEPs. These targeted interventions involve direct engagement with patrons, or staff‐mediated responses where staff are trained to identify and actively intervene with individuals exhibiting elevated risk in real time, rather than broadly applied policies or system‐wide changes. Studies were therefore included if the intervention was delivered at the individual level, either during a patron's engagement within the NEP (e.g., while intoxicated entering/leaving a venue), or through a follow‐up program targeting individuals who exhibit harmful alcohol‐related behaviours in the NEP context (e.g., emergency department during high‐alcohol hours). In contrast, studies focusing on population‐level strategies aimed at influencing the broader community, such as general venue management policies, taxation or lockouts, as well as hybrid, compliance‐based staff training approaches that provide general alcohol service guidance (e.g., responsible service of alcohol training), were excluded.

Multi‐component, hybrid interventions consisting of both individual‐level and population‐level components were excluded. Clinical interventions delivered in medical or addiction treatment settings without a direct link to NEP related behaviour were also excluded, unless participants were specifically referred following incidents or behaviour exhibited in NEPs. The primary outcome was the effectiveness (or perceived effectiveness) of these interventions in reducing alcohol‐related harms within NEPs (alcohol‐related harms including violence, injury, risky drinking or offending behaviour). Perceived effectiveness was defined as perceptions of an intervention's impact on harm, deterrence, displacement, and enforcement/compliance. Studies reporting general opinions or support for an intervention, without linking these to harm or effectiveness, were excluded. This approach ensured a focus on interventions most pertinent to nightlife environments.

### 2.3 Screening and Data Extraction

**FIGURE 1:**
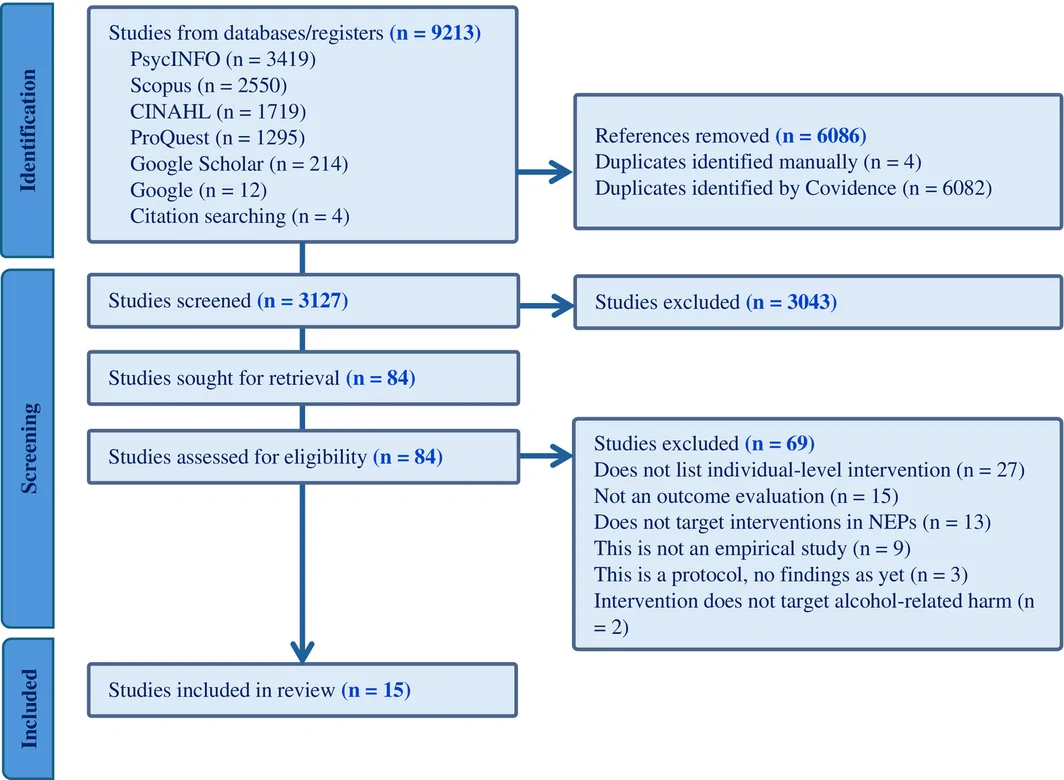
PRISMA flow diagram.

Covidence was used to screen all articles [51]. A total of 9213 articles were imported for screening. Following the removal of duplicates, 3127 articles remained which were narrowed down to 84 after excluding articles that were not relevant (reasons for exclusion can be seen in Figure 1). These articles were initially screened by the lead author (J.P.), with all studies retrieved for eligibility assessment being screened by a 2nd team member (DDA, CT, MC). All team members verified the articles in the full‐text stage. Discrepancies and disagreements between reviewers on articles were resolved through discussion. The Preferred Reporting Items for Systematic Reviews and Meta‐Analyses (PRISMA) chart is provided in Figure 1. After screening the full‐text articles, 15 studies were extracted for review. Outcome measures were documented for quantitative studies, whereas key themes and findings were noted for qualitative studies. A qualitative synthesis of existing individual‐level interventions for alcohol‐related harm in NEPs is therefore presented.

## 3 Results

### 3.1 Characteristics of Included Studies

Of the 15 studies included in the scoping review, the majority were conducted in Australia (*n* = 10), with the remainder from UK (*n* = 2), United States (*n* = 1), Denmark (*n* = 1) and Brazil (*n* = 1). These studies were categorised based on the following intervention types: exclusion strategies (*n* = 10) and behavioural strategies (*n* = 5). Quantitative studies (*n* = 10) assessed assault rates, desistance from offending, blood alcohol levels, self‐reported number of drinks consumed, or frequency of staff intervention in a bar setting. Where violence was measured, studies varied in their unit of analysis, with some studies reporting on individual‐level outcomes (e.g., reoffending following a ban) and others reporting population‐level trends (e.g., changes in assault rates at the venue or precinct level). The remaining studies were qualitative in nature (*n* = 5), where levels of perceived effectiveness for interventions were examined through interviews or surveys with key stakeholders and members of the public, providing valuable contextual insights into how the interventions were experienced, implemented and perceived within NEPs. A detailed summary of each study outlining the study details, primary measures employed and intervention effectiveness, can be found in Table 2.

**TABLE 2:** Summary of studies.

Title and citation (author)	Study design	Location	Intervention	Data source(s)	Primary measure(s)	Effectiveness	Limitations
Behavioural Strategies							
*#RU2drunk initiative* Boyd et al. [52]	Comparative study design	UK	Breathalysers	Police data, Ambulance and emergency services data, Survey data	Violent offences (aggregated level) prior to and following the breathalyser initiative in the UK (NEP). Level of public support.	In Torquay (UK), violent crime incidents decreased by 39% between 2013 and 2014, coinciding with the breathalyser initiative. The comparison site, Paignton (UK), showed a different trajectory. 75% of online survey respondents (*n* = 310) supported the breathalyser scheme.	Number of crimes recorded were small (*n* = < 25) and fluctuated over time
*QuikFix brief intervention* Hides et al. [53]	Randomised controlled trial	Australia	AUDIT test and follow up with either QuikFix (MI with individualised personality‐specific coping skills training), assessment feedback/information or MI alone	Emergency department data	Total standard of drinks consumed in the past month (individual‐level), also measured at 12 months follow up	Moderate but meaningful reduction in alcohol consumption for QuikFix compared to assessment feedback/information and MI. QuikFix group reported significantly lower standard drinks consumed than assessment feedback/information (*d* = 0.40) and MI (*d* = 0.40) at the 1‐month follow‐up, and at 12 months follow up.	No biological confirmation of self‐reported substance use. Payments may have incentivised reporting reductions in alcohol use.
*Raise the Bar* Kettrey et al. [54]	Quasi‐experimental field evaluation	United States	Bystander training for bar staff	Observations	Increased staff intervention with at‐risk patrons (individual‐level) in bars	Odds of intervention at trained bars were 2.77 times higher than at untrained bars. Staff intervention was overall relatively rare—occurring in 23% of all bar observation sessions.	Sample bias (self‐reporting). Findings were not robust (weakened when control variables were added).
*Web‐based Intervention* Sanchez & Sanudo, [55]	Randomised controlled trial	Brazil	Interviews in nightclubs (including AUDIT score and breath alcohol concentration), and follow up survey	Survey data	Prevalence of binge drinking and AUDIT score in the past month (individual‐level)	Estimated 13% reduction in the AUDIT score in favour of the intervention group compared to the control group. Average adherence to the intervention.	High rate of attrition over the 12‐month follow‐up period. Inability to compare results with other studies due to novel nature of intervention.
*Operation Drinksafe* Van Beurden et al. [56]	Non‐randomised experimental study	Australia	Brief alcohol intervention (AUDIT), blood alcohol concentration, personalised risk assessment and follow up survey	Survey data	Reductions in AUDIT scores, weekly alcohol consumption and binge drinking (individual‐level)	Effectiveness across various subgroups, particularly high‐risk drinkers, females and those with higher initial consumption. Harmful drinkers AUDIT score reduced by 29%, and binge drinking by 37%. All participants reduction in AUDIT scores (15%), weekly alcohol. consumption (13%) and binge drinking frequency (19%).	Lack of a control group—difficult to attribute observed changes solely to the intervention

Citation (author)	Study design	Location	Intervention	Data source(s)	Primary measure(s)	Effectiveness	Limitations
Exclusion Strategies							
Curtis et al. [57]	Quantitative comparative study design	Australia	Police‐imposed patron banning notice	Police data	Offences prior to and following an individual receiving a police‐issue patron banning notice in Victoria (individual‐level)	Significant increase in public order offences (*d* = 0.67) in the 2 years following a short‐term ban (up to 72 h). Lack of comprehensive coverage and oversight of the ban enforcement process limits its effectiveness as a deterrent.	Limited sample (regional city in Victoria) may affect generalisability
Farmer & Miller [58]	Qualitative, thematic analysis	Australia	Police‐imposed patron banning notice and prohibition orders	Interviews (key informants)	Levels of perceived effectiveness (enforcement)	Issues with enforcement limits the effectiveness. Data sharing practices and identification of recipients of bans in question.	Reliance on key informant perspectives limits broader applicability.
Farmer et al. [59]	Quantitative research	Australia	Police‐imposed patron banning notice and prohibition orders	Police data	Offences prior to and following an individual receiving a barring notice or prohibition order (individual‐level)	Decrease in offending after barring notices recipients (52% no further offences) and prohibition orders (58% no further offences). Multiple barring notice recipients showed a significant increase in offending post‐notice, suggesting reduced effectiveness for repeat recipients.	No control group. Small sample sizes (for some sub‐groups). Counted offence frequency only, not type or severity.
Farmer et al. [60]	Qualitative, thematic analysis	Australia	Police‐imposed patron banning notice and prohibition orders	Interviews (key informants)	Levels of perceived effectiveness (enforcement)	General support for patron bans and prohibition orders, but significant concerns related to enforcement. Technological limitations with ID scanners can limit effectiveness of enforcement.	Sample bias (key informant perspectives only).
Farmer et al. [61]	Qualitative, thematic analysis	Australia	Police‐imposed patron banning notice and prohibition orders	Panel survey (public) and interviews (key informants)	Levels of perceived effectiveness (deterrence, displacement)	Most survey respondents perceived patron banning to be moderately effective (50%, *n* = 506) or slightly effective (27%, *n* = 274). Displacement was highlighted as the most likely effect of patron ban (59%, *n* = 599). Low level of community understanding and awareness of patron banning.	Sample bias (key informant perspectives)
Farmer et al. [62]	Quantitative research	Australia	Police‐imposed patron banning notice	Police data	Offences prior to and following the introduction of patron banning notices (aggregated level)	Significant reductions (*p* < 0.001) in non‐family assault offences, assault offences recorded occurring on pathways (around licensed premises) and common assault offences	Changes to trading hours may have affected data accuracy. Inconsistent use of alcohol flag reduces reliability.
Miller et al. [63]	Qualitative thematic analysis	Australia	Police‐imposed and venue patron banning notice	Interviews (key informants)	Levels of perceived effectiveness (harm)	Key informants supported patron banning as effective for problematic individuals and enhancing safety, though banning processes were noted as inconsistent	Sample bias (key informant perspectives only)
Søgaard [64]	Qualitative research	Denmark	Police‐imposed patron banning order	Interviews (public) and police data	Levels of perceived effectiveness (harm, compliance)	All participants admitted to frequently violating their ban conditions, highlighting the ineffectiveness of patron banning orders. Negative mental health effects for some participants.	Small sample size (*n* = 10)
Stafford [65]	Quantitative research	UK	Business‐based banning sanctions (warnings and exclusions)	Offence and sanction data	Desistance from offending following warnings and exclusions in businesses (individual‐level)	Warnings (76% desistance rate) were more effective than exclusions (37% desistance rate). Variability in desistance rates based on offender characteristics and offence types. Significant portion of repeat offenders responsible for a disproportionate number of offences.	Limited detail on offender displacement
Taylor et al. [66]	Quantitative quasi‐experimental study	Australia	Police‐imposed patron banning order	Police data	Reductions in police‐recorded offences (aggregated level) in three NEPs	No evidence to suggest 10‐day police‐issued patron bans effectively reduced serious assault, common assault or good order offence trends the weekend following a ban	Data lacked detail on reasons for bans, demographics of banned individuals, ban extensions or other offences committed

Abbreviations: AUDIT, Alcohol Use Disorders Identification Test; MI, motivational interviewing; NEP, Night‐Time Entertainment Precinct; UK, United Kingdom.

### 3.2 Exclusion Strategies

#### 3.2.1 Patron Banning Effectiveness

Police‐imposed patron bans were the most prevalent individual‐focused intervention (*n* = 11) for alcohol‐related harm in NEPs (5 quantitative studies; 6 qualitative studies). The majority of these studies (*n* = 10) were Australian based (with the exception of two studies based in the UK and Denmark), reflecting settings within this context. Quantitative evaluations of patron banning explored the rate of offending prior to and after receiving a patron ban, although the outcome measures and location‐level of analyses varied considerably, as did findings on effectiveness. In Victoria, Australia, individual‐level data indicated that public order offences significantly *increased* (*d* = 0.67) among individuals who received a police‐issued patron ban in the 2 years following the ban [57]. Another study examined trends following patron banning notices at an aggregate state level, and found significant reductions in non‐family assault offences, assaults on pathways near licensed venues and common assault offences in Western Australia after the policy was implemented [62]. In contrast, Taylor et al. [66] found no evidence to suggest 10‐day police‐issued patron bans in Queensland, Australia influenced alcohol‐related harm through patterns of serious assault, common assault or good order offences.

At an individual‐level, Farmer et al. [59] examined offending patterns before and after recipients received a barring notice or prohibition order, finding that just over half of barring notice recipients (52%) and prohibition order recipients (58%) did not reoffend following the intervention. Not surprisingly, individuals who received multiple barring notices were significantly more likely to experience higher levels of offending post‐notice, while for prolific offenders (aged 18–20), the barring notice was shown to have no significant impact on their offending behaviours. Stafford [65] assessed individual‐level recidivism, comparing desistance (no further offending) with continued offending, finding stronger effects for desistance to warnings issued to patrons in response to problematic behaviour in NEPs (76%), compared to place‐based exclusions (37%). This suggests warnings may have a stronger effect on future behaviour than exclusions alone, although both influenced levels of desistance. The variation in outcome measures, ranging from individual‐level to aggregated level offence trends, makes it difficult to directly compare findings and determine the overall effectiveness of patron banning interventions across studies. Variation was also noted in the specificity of location analysed across studies, with some studies examining offences occurring within specific venues or precincts [65, 66], whereas other studies analysed trends across broader areas such as suburbs or states [57, 59, 62].

Qualitative, thematic studies evaluating the community's perceived effectiveness of patron banning (*n* = 6) reported mixed findings. Studies sampling key informants from police, licensees, venue staff and ID scanner companies reported that patron banning was viewed as an effective measure for increasing safety and reducing alcohol‐related harms [58, 60, 61], particularly for law enforcement personnel who considered it a valuable tool for immediate harm reduction [63]. In contrast, Søgaard [64] noted patron banning orders in Denmark led one patron to engage in retaliatory violence, contradicting the aims and purpose of patron banning. Individuals interviewed in this study also reported adverse impacts on their health and well‐being as a result of the patron ban, including feelings of shame, social isolation and, in some cases, self‐harm [64]. These findings suggest that patron banning may lead to unintended consequences, potentially resulting in iatrogenic harm. This contrast also underscores a divergence between perspectives: while key informants such as licensees, police, venue owners and ID scanner companies emphasised the value of patron banning for enforcement and harm reduction, members of the public raised concerns about its severe psychological and social impacts, which may lead to further violence. Some key informants (licensees and police) also acknowledged a perceived lack of community awareness of patron bans [60, 61]. This suggests that patron banning may be limited in its ability to influence behaviour or generate broader cultural change around drinking and violence in NEPs through a general deterrence effect.

Although patron banning aims to deter individuals from engaging in harmful behaviours within licensed venues [57], findings from some of the studies reviewed raise concerns regarding the potential for harm displacement [61, 65]. In the UK, for example, 66% of banned individuals were found to have committed subsequent public order and violent offences in areas outside of the location from which they were excluded, following their initial sanction [65]. This suggests that patron bans may lead to the relocation of problematic behaviour. In addition, patrons surveyed by Farmer et al. [61] perceived displacement as the most common effect of patron banning, rather than a reduction in offending or no effect at all. Limited evidence exists on the extent to which patron bans reduce total harm, especially with respect to violence occurring in nearby precincts or alternative social settings [60].

#### 3.2.2 Implementation and Enforcement Challenges

The studies included in this review suggest that the lack of enforcement of patron banning remains a critical factor contributing to non‐compliance with banning notices. Almost all qualitative studies on patron banning [57, 58, 61, 64] highlighted inconsistent enforcement as a key challenge to widespread compliance, reducing the intervention's credibility and effectiveness. In Denmark, almost all participants in one study reported occasionally violating their ban, which was considered relatively normalised, with violations often going unnoticed by authorities [64]. Consistent enforcement is a key component of deterrence‐based strategies [1]; if banned individuals do not perceive a high risk of apprehension or sanction for violating bans, this is likely to reduce the intervention's capacity to influence behaviour.

Implementation and enforcement of patron bans was also challenged by technological limitations. Qualitative studies exploring the perceived effectiveness of patron banning identified loopholes in ID scanning systems, which are employed in licensed venues within some Australian jurisdictions to detect banned patrons [58, 60, 63]. These limitations include enabling banned patrons to enter venues when scanners are not in operation (e.g., before certain hours), or in venues that do not employ scanning at all [60, 63]. This highlights the constraints of enforcement mechanisms within exclusion strategies, where inconsistent application may undermine their effectiveness in reducing harm.

#### 3.2.3 Length of Ban

Findings from the reviewed studies suggest that variability in the duration of patron banning schemes poses challenges for evaluating their effectiveness. In Victoria, Australia, Curtis et al. [57] noted that short‐term bans (up to 72 h) were likely insufficient in influencing repeat offending or anti‐social behaviour and suggested that extending the length of bans may strengthen their deterrent effect. Similarly, an evaluation of 10‐day police issued bans in Queensland, Australia found no evidence to suggest they were effective in reducing public order offences within NEPs [66]. Although Queensland have since extended their patron banning orders from 10 to 30 days (with discretion from police to extend up to 3 months) [67], short durations of patron bans may constrain their potential to induce sustained behavioural change. The impact and efficacy of short‐term, or even long‐term, bans remain under‐researched, despite their potential to significantly affect civil liberties. This highlights a critical gap in our understanding of the broader implications and effectiveness of patron banning in reducing alcohol‐related harm in NEPs.

#### 3.2.4 Summary

Ultimately, there is mixed evidence to support the effectiveness of patron bans and there is a limited understanding of how these interventions may impact violence and other alcohol‐related harms in NEPs. The lack of robust empirical evidence and the potential for displacement and other social or psychological harms point to the limitations of patron bans in addressing the underlying drivers of alcohol‐related harm, raising questions about their role as a meaningful long‐term solution to violence and harm within NEPs.

### 3.3 Behavioural Strategies

Interventions that could be described as behavioural strategies (*n* = 5) comprised the remainder of studies identified.

#### 3.3.1 Brief Intervention and Personalised Screening Effectiveness

Brief screening and intervention strategies were associated with measurable reductions in alcohol consumption, particularly among high‐risk drinkers (*n* = 3). Across BI studies, MI and personalised risk assessments consistently led to decreased AUDIT scores, lower weekly alcohol intake and reduced binge drinking frequency [53, 55, 56]. A consistent theme across these studies was the value of personalised feedback in promoting self‐reflection and encouraging positive behavioural change, such as reductions in risky drinking. For instance, Operation Drinksafe [56] recorded a 15% reduction in AUDIT scores, a 13% decrease in weekly alcohol consumption, and a 19% reduction in binge drinking frequency following BI in licensed venues. Similarly, nightclub patrons who received web‐based personalised feedback had a 13% reduction in AUDIT scores at 6 months post‐intervention [55]. The QuikFix trial, which combined brief screening with MI, also demonstrated a moderate but meaningful impact, leading to greater reductions in alcohol consumption at 12 months for patrons compared to assessment feedback/information for MI alone [53]. Collectively, these findings suggest that tailoring interventions to individual risk levels enhances their effectiveness and engagement in reducing risky drinking behaviours.

#### 3.3.2 Breathalyser Use and Bystander Intervention Effectiveness

One study examined the use of breathalysers for patrons upon entry to licensed premises, with findings suggesting it is a promising strategy for positive behavioural change in patrons. Breathalysers employed by door staff at licensed premises in Torquay, UK, to assess patrons who appeared intoxicated or posed a potential risk coincided with a 39% reduction in violent crime incidents between 2013 and 2014 compared to a comparison site [52], suggesting real‐time feedback may moderate drinking behaviour. Although Paignton, UK, was included as a comparison site, they also implemented breathalysers, meaning this was not a controlled study. Crime rates in these locations were also relatively low [52]; however, the study shows promising results for incorporating breathalysers into harm reduction strategies within licensed venues.

Only one study examined bystander awareness intervention for bar staff. Raise the Bar, a targeted prevention program that trained bar staff to intervene when witnessing risk factors for sexual assault, led to positive behaviour effects including staff being more likely to engage in increased check‐ins with patrons, interrupting risky situations and refusing service to intoxicated individuals, compared to bars that did not receive any training [54]. However, staff intervention rates remained relatively low overall, due to staff's limited situational awareness, discomfort with confrontation, and a lack of confidence in applying the intervention strategies. Additionally, the observed effects attenuated when control variables, such as contextual factors, were introduced, revealing limited significance of this study's findings [54].

#### 3.3.3 Effectiveness for Specific Sub‐Groups

Within the behavioural intervention category, the Operation Drinksafe BI [56] found that while reductions in AUDIT scores, weekly alcohol consumption, and binge drinking were observed across all participants, greater reductions across these measures were seen among high‐risk drinkers, particularly those with higher initial alcohol consumption. Similarly, Sanchez and Sanudo [55] found significant reductions in both AUDIT scores and binge drinking prevalence among high‐risk groups in both the control and the intervention subgroups, compared to the low‐risk group who, after 6 months, had increased AUDIT scores. These studies suggest that individuals with higher‐risk drinking levels may benefit most from more targeted or intensive strategies. It supports the notion that individuals with varying levels of risk respond differently to interventions, and that tailoring interventions based on initial consumption levels may yield more meaningful behavioural change.

#### 3.3.4 Primary Outcome Measures

Although the reviewed studies note that BI have shown some effect in reducing individual alcohol consumption, only one study has examined their impact on broader alcohol‐related harms, such as violence and injury. Boyd et al. [52] analysed the impacts of a police‐led breathalyser initiative on violent offences by examining recorded crime data prior to and following the scheme. Noticeable reductions were observed in levels of reported violent crime; however, as this study noted many methodological limitations including small offence numbers and absence of repeated measures for the same individuals [52], the generalisability and evidence for broader harm reduction remains unclear. The other four studies focusing on behavioural change [53, 54, 55, 56], did not examine the impact that alcohol consumption or binge drinking has on rates of assault, violence or disorderly behaviour in nightlife environments. Exclusion strategy studies in comparison were solely quantitatively assessed in terms of violent offending outcomes. This highlights a key gap in the current research, suggesting that while BI may reduce levels of risky drinking, their effect on measures of alcohol‐related crime and harm is rarely assessed and remains uncertain.

#### 3.3.5 Implementation Challenges

Similarly to patron banning, some studies in the behavioural strategies category faced difficulty with effective implementation, limiting their overall potential as an effective intervention to target alcohol‐related harm in NEPs. For instance, Sanchez and Sanudo [55] web‐based study had a high attrition following the BI intervention due to loss of participants at different stages in the intervention and challenges in accessing the internet by some participants. Limited staff confidence in intervening in bystander scenarios [54] also revealed challenges with implementing bystander strategies. These studies are also relatively novel, relying on quasi‐experimental designs [54], preliminary trials [52, 53, 55] or non‐randomised designs [56]. Such limitations make it difficult to fully assess the practical challenges of implementing these interventions and indicate the need for more rigorous research.

## 4 Discussion

The findings of this review suggest that while some individual‐level interventions show promise in reducing alcohol‐related harm in NEPs, challenges in effective implementation and substantial variation in study designs and outcome measures make it difficult to establish their overall effectiveness. Exclusion strategies, such as patron banning, demonstrated some effectiveness in reducing offending [59, 62, 65], but overall revealed limited long‐term impact in reducing violence and other harms, which may be in part due to inconsistent enforcement [57, 58, 61, 64] and harm displacement [61, 65]. Stakeholder views were divided, with key informants from a regulatory background noting the benefits of patron bans [60, 63] while members of the public raised concerns around the negative mental health impacts [64].

Behavioural interventions, particularly brief, personalised strategies or feedback‐based approaches, more consistently demonstrated effectiveness in reducing alcohol consumption over longer periods of time. These interventions were associated with reductions in self‐reported alcohol consumption [53, 55] and risky drinking, particularly among high‐risk individuals [56]. However, besides one study analysing rates of violence following the introduction of breathalysers in licensed venues [52], behavioural studies primarily measured drinking behaviours, overlooking outcomes related to violence or broader harm. Their effectiveness in reducing aggressive or antisocial behaviour therefore remains unclear.

Across both intervention types, enforcement and implementation challenges emerged as recurring themes. Inconsistent implementation, enforcement [57, 58, 61, 64] and technological challenges to monitoring of patron bans [58, 60, 63] were believed to limit their potential effectiveness. Limited staff confidence in intervening in bystander scenarios [54] may undermine the efficacy of these interventions. Another BI also had low adherence rates due to practical and operational barriers [55]. Many behavioural strategy studies were methodologically limited, being observational in nature, non‐randomised and having a small sample size [52, 53, 54, 55, 56]. These challenges point to practical barriers that can significantly hinder the impact and effectiveness of such interventions in reducing harm for individuals within NEPs.

There is limited research on the characteristics of individuals who offend in NEPs and how interventions affect different risk groups. Among exclusion strategy studies, Stafford [65] utilises individual offence‐level data to contextualise patterns of alcohol‐related harm, while Farmer et al. [59] differentiate individuals by offending sub‐groups (single offence, multiple offences, prolific offenders)—all other exclusion strategy studies predominantly focus on difference by offence type, such as violent versus non‐violent offences. This lack of stratification limits our understanding of how different types of individuals (based on their risk profiles) may respond to the same intervention. Without this distinction, it is challenging to assess whether patron banning is more effective for high‐risk individuals or for those at lower risk of causing harm, potentially resulting in misallocated resources or ineffective interventions. In comparison, behavioural strategies were identified as effective for those with more serious, risky drinking behaviours [53, 55, 56]. Understanding this variation is crucial for ensuring that interventions are appropriately aligned with the needs and risk profiles of individuals who engage in alcohol‐related violence. Without an understanding of how individuals respond to interventions based on their risk profiles or offending patterns, the ability of individual‐level interventions to have discernible impacts on the level of alcohol‐related violence and harm in NEPs remains unclear.

Additionally, all studies in this review, apart from Stafford [65] and Farmer et al. [59], failed to examine or comment on the longitudinal patterns of violent behaviour among individuals in NEPs, such as repeat offending, escalation, or offending persistence over time. Individuals who displayed repeated problematic behaviour, and who were subject to repeat notices, showed a worsening of offending behaviour in the subsequent period, suggesting that patron bans may work better for individuals engaged in one‐off or short‐term problematic behaviours in NEPs, and perhaps worsen outcomes for individuals whose drinking or associated antisocial behaviour is more entrenched [59]. Although Farmer et al. [59] study notes patron bans had limited impact on individuals with an established pattern of offending, the small sample size of this sub‐group limits how confidently these findings can be generalised. More broadly, it remains unclear whether violent behaviour in NEPs may be situational or habitual. Individuals with chronic substance use issues or prior criminal histories, for instance, may not experience the same outcomes from patron banning as those who engage in one‐off incidents of disorderly behaviour. The current evidence base is therefore underdeveloped, with limited research assessing sustained behavioural change, repeat offending or differential impacts across different subgroups.

### 4.1 Policy Implications

This study addresses a key knowledge gap in the literature, exploring how individually targeted interventions can address key risk factors that could be used to tackle alcohol‐related violence in NEPs. Exclusion strategies like patron banning intend to produce a deterrent effect or serve symbolic functions in reinforcing behavioural norms. However, without consistent enforcement and broader public awareness, their impact can be limited. Additionally, without clear evidence on the optimal length of a patron ban or an understanding of which individuals benefit most from such interventions, their potential to drive long‐term behaviour change appears limited [57]. It is not clear how these interventions impact high‐risk or repeat offenders who may drive a disproportionate amount of harm in NEPs. Policymakers should be cautious in relying on patron banning alone as an intervention to reduce long‐term, alcohol‐related harm in NEPs.

There is promising evidence to suggest that behavioural strategies, such as BI, are effective for high‐risk drinkers, particularly those consuming alcohol at hazardous or risky drinking levels. However, further research employing rigorous methodologies, including randomised designs, larger samples and accounting for pre‐existing risk factors, is needed to determine their impact on alcohol‐related harm. The relaxation of liquor licensing regulations internationally may lead governments to consider an expansion of person‐centred, individual‐level interventions. Therefore, it is timely to consider how the standard and quality of these interventions can be enhanced to ensure that they are effective at preventing harm and increasing safety for the wider community. There is also a lack of evidence on which individuals produce the majority of violent crime in NEPs, their patterns of offending and the specific needs and risks of persistent violent people in NEPs. A better understanding of persistently violent and problematic individuals in these settings would enhance the targeting and long‐term effectiveness of individual‐level interventions.

### 4.2 Limitations

This scoping review examined alcohol‐related harm within NEPs, excluding interventions that addressed harmful alcohol use in broader contexts, such as healthcare settings, festivals, or private settings. Although interventions in NEPs are relevant to reducing alcohol‐related harm, limiting the focus purely to these settings narrowed the scope of eligible articles included in the review. This review also analysed interventions at an individual‐level, excluding some studies that might influence individual behaviour indirectly, but as the focus was on purely individual interventions, it was outside of the scope of this review. Additionally, many of the studies reviewed were relatively novel interventions with quasi‐experimental studies or observational research study designs. As a result, strong conclusions about the effectiveness or long‐term impact on behaviour from individual‐level interventions cannot yet be drawn as the evidence base remains underdeveloped.

## 5 Conclusion

Although there are a small number of promising studies that have examined behavioural strategies, the current evidence base lacks the methodological rigour and consistency needed to draw firm conclusions about their effectiveness in reducing alcohol‐related harm in NEPs. Patron bans in general have mixed evidence in terms of their impacts on violent offending and limited evidence of long‐term impact. Both exclusion strategies and behavioural approaches tend to overlook key differences in risk and behaviour, such as high‐risk individuals with entrenched patterns or low‐risk individuals whose offending is more situational. Future research should prioritise identifying distinct profiles of individuals who engage in alcohol‐related aggression or harm to explore the potential benefits of targeting higher‐risk or higher‐harm individuals with more tailored strategies. Well‐designed, methodologically robust studies are needed to establish the efficacy of such strategies, particularly those that measure outcomes beyond alcohol consumption alone, including broader indicators of violence and harm.

## Author Contributions

Each author certifies that their contribution to this work meets the standards of the International Committee of Medical Journal Editors.

## Funding

D.D.A. is supported by an Australian Research Council Discovery Early Career Researcher Award (DE230101466, ‘Violent offenders in the night‐time economy: Building the evidence’ 2023–2026). J.P. is supported through a scholarship as part of the Australian Research Council Discovery Early Career Researcher Award (DE230101466, ‘Violent offenders in the night‐time economy: Building the evidence’ 2023–2026). M.M. is supported by an Australian Research Council Discovery Early Career Researcher Award (DE240100066. 2024–2026). The funding source had no role in study design, analysis and interpretation of data, preparation of manuscript, or any decision to submit the manuscript.

## Conflicts of Interest

The authors declare no conflicts of interest.

## Data Availability

Data sharing not applicable to this article as no datasets were generated or analysed during the current study (scoping review).
